# Stem cells in Dentistry: knowledge and attitude of Nigerian Dentists

**DOI:** 10.1186/1472-6831-13-27

**Published:** 2013-06-15

**Authors:** Matthew Asizide Sede, Ochuwa Audu, Clement Chinedu Azodo

**Affiliations:** 1 Department of Restorative Dentistry, University of Benin, Benin-City, Nigeria; 2 Department of Restorative Dentistry, University of Benin Teaching Hospital, Benin-City, Nigeria; 3 Department of Periodontics, New Dental Complex, University of Benin Teaching Hospital, P.M.B. 1111 Ugbowo, Benin-City, Edo State 300001, Nigeria

**Keywords:** Stem cells, Dentistry, Knowledge, Attitude

## Abstract

**Background:**

Several controversies exist about the methods of harvesting and eventual utilization of stem cells in Medicine and Dentistry. The objective of the study was to investigate the awareness, attitude and knowledge of the use of stem cells in Dentistry among Nigerian Dentists.

**Methods:**

This descriptive, cross-sectional study was conducted among dentists selected from both private and public health sectors, in some of the major cities in Nigeria.

**Results:**

The majority of the participants were ≤35 years in age, male, Pentecostal Christians, possessed a postgraduate qualification, had practiced for ≤5 years and were specialists or specializing. In this study, 153(81.0%) of the participants reported awareness about the use of stem cells in dentistry which was significantly associated with qualification and type of practice. Most of the respondents 114 (60.3%) had a poor knowledge of the use of stem cells in Dentistry. This was significantly associated with type of practice and awareness about stem cell use in dentistry but binary logistic regression showed awareness as the only determinant of knowledge. About three-quarters 142 (75.1%) of the participants exhibited positive attitude towards stem cell use. This had a positive non-significant association with knowledge and reported awareness.

**Conclusion:**

Data from this study revealed a high level of awareness, positive attitude to and poor knowledge of the use of stem cells in Dentistry among a cross section of Nigerian Dentists.

## Background

Stem cells are cells that are capable of self-replication and differentiation into at least two different cell types [1]. The self-renewal characteristics of stem cells enables them to go through numerous cycles of cell division whilst maintaining the undifferentiated state and also the ability to proliferate and differentiate into multiple mature cell types [2]. In theory, stem cells can therefore divide without limit to replenish any other cell type and also function as part of the body’s repair system [3].

There are primarily two types of stem cells namely embryonic stem cells (ESCs) and adult stem cells (ASCs) or somatic stem cells. The embryos from which the human embryonic stem cells are derived are four to five days old - “blastocyst stage” [4] and their use is controversial due to the need to destroy an embryo to harvest them. Embryonic stem cells are pluripotent and the most versatile stem cells with an unlimited capacity to proliferate and differentiate thereby facilitating their attraction as sources of stem cells [5]. However their drawback lies in the likelihood of neoplastic changes, if the proliferation and differentiation of the cells is not carefully controlled. Adult stem cells do not require embryo destruction [6] and are not subject to the ethical controversy that is associated with embryonic stem cells [7]. Although adult stem cells lack the potency of their embryonic counterparts, they have been used successfully to treat disease. In comparison with embryonic stem cells, adult stem cells possess only multipotent differentiation capacity [8]. Some documented sources of adult stem cells include umbilical cord blood, amniotic fluid, bone marrow, adipose tissue, brain, teeth, skin and urine [9-12].

Stem cells, when directed to differentiate into specific cell types, offer the possibility of a renewable source of replacement cells and tissues to treat conditions such as spinal cord injury, stroke, heart disease, diabetes, arthritis, radiation-induced tissue damage, Parkinson and Alzheimer’s diseases [13-20]. Stem cells have also shown potentials in reversing the effects of age, offering a possible ‘cure’ for aging altogether [21]. Stem cells have also been reportedly used to reverse blindness [22] and are currently being investigated for use in the treatment of graft-versus-host disease, Crohn’s and lupus, based on their ability to modulate the immune system [23,24] and in the screening of potential anti-tumor drugs with promising data arising from animal-testing [3].

Dental Stem Cells (DSCs) derived from tooth structures are adult stem cells that have captured the attention of researchers over the past decade [5]. These stem cells are readily accessible and can be obtained and stored for future use through minimally invasive procedures [25]. Just as DSCs have the potential for use throughout the body, stem cells from extra-oral sites may be used for regenerating dental tissue [5]. Roots of teeth and periodontal ligaments have been regenerated from dental stem cells [26]. Cells from tooth buds have been differentiated into a small tooth structure and transplanted in vivo [27]. Mesenchymal stem cells from the pulp and the follicle associated with third molars are under investigation for possible dentine [28] and enamel [29] production respectively. Clinical studies have been conducted using stem cells for alveolar ridge augmentation [30,31], reconstruction of a resected mandible [32] and generation of a human-shaped temporomandibular joint [33-36].

Despite the well known potentials of stem cell research and therapies in dentistry, several controversies dog the methods of harvesting and eventual utilization of stem cells. Some European Union member states have a restrictive approach on embryonic stem cell research because of religious, cultural and historical reasons [37].

The problems with stem cell use are diverse, including, but not limited to lack of basic knowledge of stem cells, ethical and religious issues concerning their use [37-41] and foremost among them is the moral concern regarding embryo destruction [38-41]. Other issues include the possibility of human cloning, the potential exploitation of embryo and egg donors, as well as the questions raised by the new alternative techniques for obtaining stem cells [40].

The field of Dentistry is not spared of these controversies and knowledge dearth that envelopes the use of stem cells. A literature search revealed that there is dearth of information relating to the use knowledge of and attitude to the of stem cells in Medicine and Dentistry. A practitioner survey of opinions toward regenerative endodontics in the United States showed that 96% of participants thought more regenerative therapies should be incorporated into dental treatments, with findings which suggested that endodontists were supportive and optimistic about the future use of regenerative endodontics [42]. The objective of this study was to investigate awareness, attitude and knowledge of the use of stem cells in Dentistry among Nigerian dentists.

## Methods

This descriptive, cross-sectional study was conducted among consenting dentists from both private and public health sectors, in randomly selected major cities in the southern part of Nigeria between February and June, 2012. The study protocol was reviewed and approved by the Research and Ethics Committee of University of Benin Teaching Hospital, Benin City, Edo State, Nigeria. Using the Cochran (1977) [43] statistical formula, the minimum sample size was calculated, but a 10% was added to amount for non response and incomplete questionnaires giving a total of 191 but 200 dentists were finally recruited for this study.

The survey was carried out using a self-administered, anonymous, twenty nine item, structured, hand and mail-delivered questionnaire. The questionnaire was pretested amongst selected dentists before the study commenced, to identify any problem areas in the questionnaire and to make appropriate alterations, as adjudged necessary. The content validity was confirmed from an in-depth literature search and a review of different questions asked in several continuing dental education end of course assessments which were accessed on the internet. The questionnaire was divided into three broad sections:

### Section A

Questions here were close-ended and dealt with socio-demographic variables, awareness and sources of information of stem cell use in Dentistry. The socio-demographic variables included age, gender, religion, field of practice, number of years in practice, designation and highest professional qualification.

### Section B

Here, structured questions were used to assess the general knowledge of stem cells- their characteristics, types, potency, plasticity, general sources, dental sources and potential uses in dentistry. Two questions were included to elicit the knowledge of ethical problems surrounding stem cell use and stem cell banking respectively.

All questions in this section were close ended, with instructions to participants to select answers, as appropriate. Questions were sourced, with some modifications, from the questions asked in the end of course assessment following a continuing dental education course organized by the Academy of Dental Therapeutics and Stomatology, United States of America, in 2007 [7].

### Section C

This section was made up of twelve items and a four-point Likert scale (“strongly agree”, “agree”, “disagree” and “strongly disagree”) was used to assess the response of the participants as regard their attitude to the use of stem cells in Dentistry. Opinions expressed were drawn from several articles, journals and internet blogs which dealt with the controversial and sensitive issues surrounding stem cell collection, utilization and human cloning. These issues revolved around ethics, morality and the moral status of the embryo, religion, motives, trust and fear. The negative opinions were intermingled with the positive ones, to give room for a wide range of attitudes to be expressed.

Data from questionnaires were manually scored and graded, coded and finally entered into Statistical Package for Social Sciences (SPSS) version 16.0 for data analysis. All data collected was subjected to descriptive and inferential statistics to generate frequencies, percentages and Chi-square values at a significance of P < 0.05. Logistic regression was also performed on the collated data to ascertain the determinant of knowledge regarding stem cells among the participants.

The knowledge section was graded in the range of zero (0) to thirty three (33), with 0–11 being poor knowledge, 12–22, being fair knowledge and 23–33 good knowledge. A total of twelve questions measured attitude to stem cell use in the questionnaire. Scoring for each question was a maximum score of four (4) and minimum score of one (1) giving a cumulative minimum score of twelve (12) and maximum score of forty-eight (48). A score of 12–30 indicated an overall negative attitude while a score of 31–48 indicated an overall positive attitude to stem cell research and therapy.

## Results

The majority of the participants were 35 years old or less, male, Pentecostal Christians possessed an additional postgraduate qualification, had practiced for 5 years or less and were specialists or specializing (Table 1). About one-third of the participants were non-specialist. Oral and Maxillofacial Surgery, Orthodontics, Restorative and Oral Pathology were the four leading fields of practice among participants that were specialist or specializing (Table 2).

**Table 1:** Demographic characteristics of the participants

**Characteristics**	**Frequency**	**Percent**
Age (years)		
≤35	133	70.4
>35	56	29.6
Gender		
Male	124	65.6
Female	65	34.4
Religion		
Pentecostal Christianity	107	56.6
Orthodox Christianity	63	33.3
Islam	16	8.5
Unspecified	3	1.6
Qualification		
Basic	55	29.1
Additional	134	70.9
Years of experience		
≤5	104	55.0
>5	85	45.0
Type of practice		
Non specializing	81	42.9
Specializing/specialist	108	57.1
Total	189	100.0

**Table 2:** Field of practice among the participants

**Field**	**Frequency**	**Percent**
Non specialist	67	35.4
Oral & maxillofacial surgery	37	19.6
Orthodontics	18	9.5
Restorative	15	7.9
Oral pathology	15	7.9
Community dentistry	11	5.8
Prosthodontics	8	4.2
Oral medicine	8	4.2
Pediatric dentistry	7	3.7
Periodontology	3	1.6
Total	189	100.0

**Figure 1:**
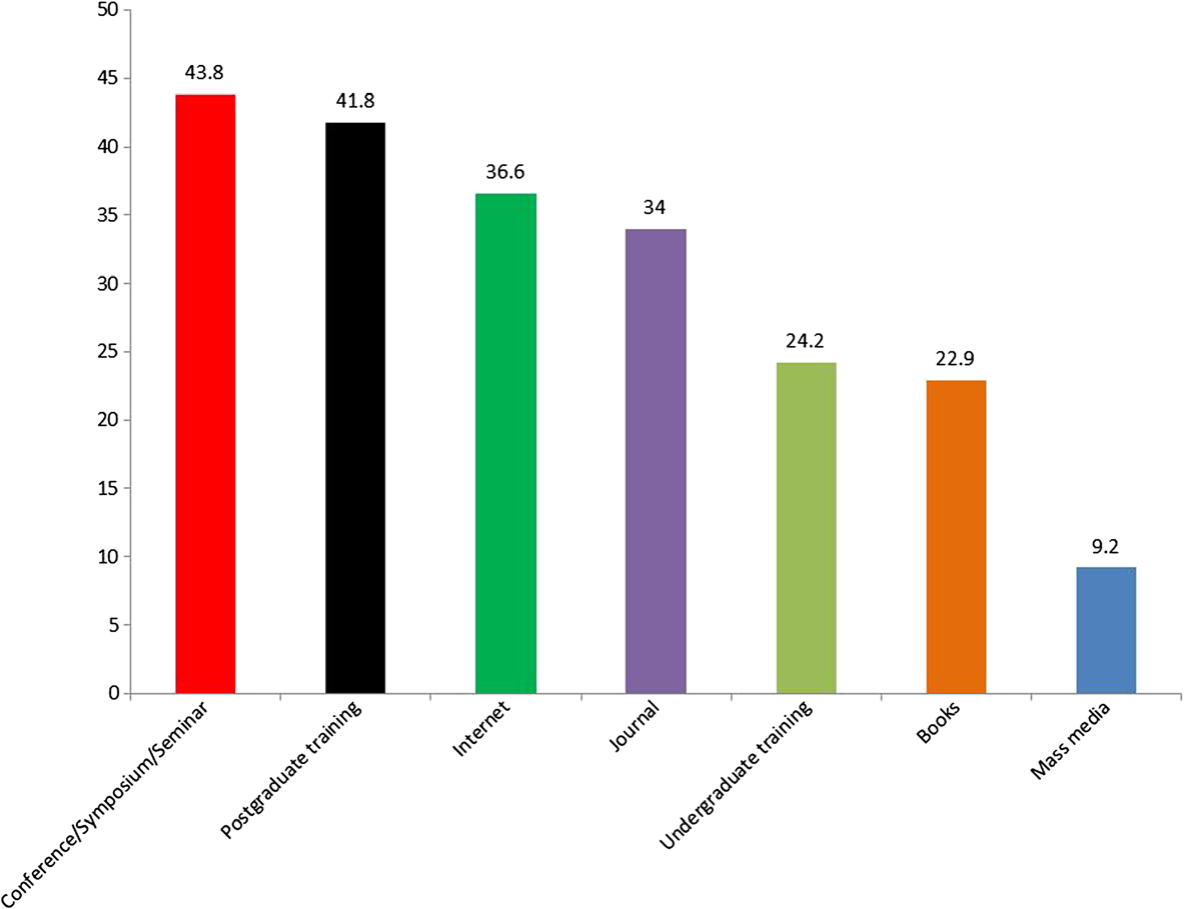
Sources of information regarding stem cell among the participants.

The leading source of information regarding stem cells was conference/symposium/seminar among 67 (43.8%) of the participants while mass media 14 (9.2%) was the least source of information (Figure 1). Most of the respondents, 114 (60.3%) had poor knowledge about the use of stem cells in dentistry.

In this study, awareness about stem cell use in dentistry was reported by 153 (81.0%) of the participants. This awareness was found to be higher among older participants (aged >35 years), muslim, males participants, more experienced in terms of years of practice (>5 years), those that possessed postgraduate qualification and those participants who were specialist or specializing. However, it was only qualification (P = 0.024) and type of practice (P = 0.001) that were significantly associated with awareness about stem cell use in dentistry (Table 3).

**Table 3:** Awareness concerning stem cell use in dentistry among the participants

**Awareness**			
	**Yes**	**No**	**P-value**
	**n (%)**	**n (%)**	
Characteristics			
Age (years)			0.787
≤35	107 (80.5)	26 (19.5)	
>35	46 (82.1)	10 (17.9)	
Gender			0.072
Male	105 (84.7)	19 (15.3)	
Female	48 (73.8)	17 (26.2)	
Religion			0.625
Pentecostal Christianity	84 (78.5)	23 (21.5)	
Orthodox Christianity	52 (82.5)	11 (17.5)	
Islam	14 (87.5)	2 (12.5)	
Years of practice			0.119
≤5	80 (76.9)	24 (23.1)	
>5	73 (85.9)	12 (14.1)	
Qualification			0.024
Basic	39 (70.9)	16 (29.1)	
Additional	114 (85.1)	20 (14.9)	
Type of practice			0.001
Non specializing	57 (70.4)	24 (29.6)	
Specializing/specialist	96 (88.9)	12 (11.1)	

The knowledge about the use of stem cells in dentistry was higher among the older (aged >35 years), muslim, female participants, who were more experienced in terms of years of practice (>5 years). Those who possessed a postgraduate qualification and were specialists or in the process of specializing, reported awareness about the use of stem cells in dentistry and expressed positive attitudes towards the use of stem cells. However, it was the type of practice (P = 0.030) and awareness about the use of stem cells in dentistry (P = 0.002) that were significantly associated with knowledge of the use of stem cells (Table 4).

**Table 4:** Knowledge about stem cell among the participants

**Knowledge**			
	**Good/fair**	**Poor**	**P-value**
	**n (%)**	**n (%)**	
Characteristics			
Age (years)			0.060
≤35	47 (35.3)	86 (64.7)	
>35	28 (50.0)	28 (50.0)	
Gender			0.949
Male	49 (39.5)	75 (60.5)	
Female	26 (40.0)	39 (60.0)	
Religion			0.710
Pentecostal Christianity	42 (39.3)	65 (60.7)	
Orthodox Christianity	25 (39.7)	38 (60.3)	
Islam	8 (50.0)	8 (50.0)	
Qualification			0.211
Basic	18 (32.7)	37 (67.3)	
Additional	57 (42.5)	77 (57.5)	
Years of practice			0.030
≤5	34 (32.7)	70 (67.3)	
>5	41 (48.2)	44 (51.8)	
Type of practice			0.122
Non specializing	27 (33.3)	54 (66.7)	
Specializing/specialist	48 (44.4)	60 (55.6)	
Awareness of stem cell use in dentistry			0.002
Yes	69 (45.1)	84 (54.9)	
No	6 (16.7)	30 (83.3)	
Attitude towards stem cell use			0.823
Positive	57 (40.1)	85 (59.9)	
Negative	18 (38.3)	29 (61.7)	

Logistic regression showed that awareness was the only determinant of knowledge about the use of stem cells. The participants who reported awareness about use of stem cells in dentistry had more knowledge about stem cell use (P = 0.003) (Table 5). About three-quarters-142 (75.1%) of the participants exhibited a positive attitude towards stem cell use. There was a non significant association between attitude towards the use of stem cells with age, gender, religion, years of practice, qualification, type of practice and awareness about the use of stem cells in dentistry (Table 6).

**Table 5:** Determinants of knowledge about stem cell among the participants

**Characteristics**	**O.D**	**95% C.I**	**P-value**
Age (years)			
≤35	1	-	-
>35	0.657	0.289-1.496	0.317
Gender			
Male	1	-	-
Female	0.852	0.442-1.643	0.633
Religion			
Islam	1	-	-
Pentecostal Christianity	0.750	0.235- 2.394	0.627
Orthodox Christianity	0.941	0.482-1.837	0.858
Qualification			
Basic	1	-	-
Additional	0.866	0.370-2.162	0.757
Years of practice			
≤5	1	-	-
>5	0.713	0.347-1.683	0.440
Type of practice			
Non specializing	1	-	-
Specializing/specialist	1.122	0.448-2.808	0.806
Awareness about stem cell use in dentistry			
No	1	-	-
Yes	0.235	0.089-0.617	0.003

**Table 6:** Attitude towards stem cell application among the participants

**Attitude**			
	**Positive**	**Negative**	**P-value**
	**n (%)**	**n (%)**	
Characteristics			
Age (years)			0.478
≤35	98 (73.7)	35 (26.3)	
>35	44 (78.6)	12 (21.4)	
Gender			0.315
Male	96 (77.4)	28 (22.6)	
Female	46 (70.8)	19 (29.2)	
Religion			0.904
Pentecostal Christianity	80 (74.8)	27 (25.2)	
Orthodox Christianity	49 (77.8)	14 (22.2)	
Islam	12 (75.0)	4 (25.0)	
Years of practice			0.191
≤5	82 (78.8)	22 (21.2)	
>5	60 (70.6)	25 (29.4)	
Qualification			0.083
Basic	46 (83.6)	9 (16.4)	
Additional	96 (71.6)	38 (28.4)	
Type of practice			0.159
Non specializing	65 (80.2)	16 (19.8)	
Specializing/specialist	77 (71.3)	31 (28.7)	
Awareness			0.192
Yes	118 (77.1)	35 (22.9)	
No	24 (66.7)	12 (33.3)	

## Discussion

In this study, the majority of the participants were aged 35 years or less, male, Pentecostal Christians, possessed additional postgraduate qualification(s), had practiced for 5 years or less and were specialist or specializing which reflected the dominant status of actively practicing dentists in the major cities in the Southern part of Nigeria. The increased specialist training opportunity due to the opening of new dental schools and the improvement in the remuneration of specializing doctors in Nigeria in recent times may have been the attraction thereby increasing the number of dental specialists or specializing dentists reported in this study. Oral and Maxillofacial Surgery was the leading field of practice among participants that are specialist or specializing. The leading specialty reported in this study is in tandem with findings of studies on career choices among Nigerian dental students [44].

In Dentistry, stem cells have the potential of providing permanent cures for all lesions of pulpal or periodontal origins. Congenital and acquired intra and extra-oral soft and hard tissue defects could also be effectively managed or treated with stem-cell based approaches and future tooth replacements in the form of ‘ a new natural tooth’ now seem like a possibility.

In this study, awareness of the use of stem cells in dentistry was reported by 153 (81.0%) of the participants. This awareness about the use of stem cells in dentistry was higher among the older muslim and male participants (aged >35 years), who were more experienced in terms of years of practice (>5 years), those that possessed a postgraduate qualification and those participants who were specialist or in the process of specializing. However it was essentially qualification and type of practice that were significantly associated with awareness about stem cell use in dentistry. These findings can be explained by the fact that conference/symposium/seminar, postgraduate training, internet and journals (the leading sources of information regarding stem cells among the participants) are the dominant mode of learning among those participants who are specialist or specializing and had an additional postgraduate qualification. The low contribution of undergraduate training to awareness explained why awareness about stem cell use in dentistry was higher among older participants (aged >35 years) and participants more experienced in terms of years of practice (>5 years). The higher awareness about use of stem cells in dentistry among males may be explained in two folds. Firstly, it may be due to the dominance of males in postgraduate training. Secondly, the greater information seeking ability characteristics of males due to their attribution of success to external causes in comparison to females leads to their conference/symposium/seminar attendance [45].

An overall good/fair knowledge about the stem cells in the study was noted among 75 (39.7%) of the participants. This overall good/fair knowledge about the use of stem cells was significantly higher among specialist or those specializing and participants that reported awareness about use of stem cell in dentistry; a binary logistic regression showed that awareness was the only determinant of knowledge about the use of stem cells. The positive effects of awareness about issues on knowledge have been consistently reported [46] thereby explaining the effect of awareness on knowledge in this study as variables on higher awareness except for gender were same for knowledge about stem cells. Gender differences were found to be in the knowledge distribution, with females having more fair/good knowledge about stem cells despite lower awareness of the use of stem cells in dentistry may be linked with the fact that female dentists have higher tendencies to accept and pay more attention to details in new trends while males are more likely to resist them thereby lessening their overall knowledge. The reported male preference for rational evaluation and logical learning style compared to female elaborative processing learning style may be a contributory explanation [47].

In this study, three-quarters 142 (75.1%) of the participants exhibited a positive attitude towards stem cell use. Similarly, a recent study [48] among a group of dental residents in the United States of America revealed considerable support for stem use in dentistry though with some reservation about the possible health hazards. This high level of positive attitude can be explained by the high level of awareness about stem cell noted in this study. This is based on the fact that expressed positive attitude to stem cell had a positive non-significant association with knowledge about stem cell and reported awareness about stem cell use in dentistry.

Religious reservations concerning stem cell use have been documented in the literature [37,40,41]. However, religion was not a stable and significant factor in this study because a more positive attitude towards stem cell was found among orthodox Christians meanwhile a non-statistically significant higher awareness and an overall fair/good knowledge was found among Muslim participants. The high awareness, poor knowledge and positive attitude towards stem cell use may be responsible for this finding. The handling of religious issues with care among professionals in comparison with the general public may be contributory.

## Conclusion

Data from this study revealed a high level of awareness, positive attitude to and poor knowledge of the use of stem cells in Dentistry among a cross section of Nigerian Dentists. Awareness about the use of stem cells in dentistry was a significant determinant of knowledge about the use of stem cells in this study. The improvement of knowledge about the use of stem cells in dentistry is achievable through heightened awareness creation on the issue. It can also be concluded that the use of stem cells in dentistry is acceptable to Nigerian Dentists.

## Competing interests

The authors declare that they have no competing interests.

## Authors’ contributions

SMA conceived the study, participated in its design and coordination, and helped to draft the manuscript. OA made substantial contributions to conception and design, acquisition of data and helped to draft the manuscript. ACC contributed substantially to design, data analysis and interpretation and helped to draft the manuscript. All authors read and approved the final manuscript.

## Pre-publication history

The pre-publication history for this paper can be accessed here:

http://www.biomedcentral.com/1472-6831/13/27/prepub
